# Feasibility and acceptability of technology-based caregiver engagement strategies delivered in a summertime childhood obesity prevention intervention: results from an internal pilot of the Camp NERF (Nutrition, Education, Recreation, and Fitness) study

**DOI:** 10.1186/s40814-018-0340-2

**Published:** 2018-09-27

**Authors:** Laura C. Hopkins, Mary Fristad, Jacqueline D. Goodway, Bernadette Melnyk, Ihuoma Eneli, Chris Holloman, Julie A. Kennel, Alison Webster, Amy R. Sharn, Carolyn Gunther

**Affiliations:** 1 Interdisciplinary PhD Program in Nutrition, Department of Human Sciences, Human Nutrition Program, The Ohio State University, 262B Campbell Hall, 1787 Neil Avenue, Columbus, OH 43210 USA; 2 Department of Psychiatry and Behavioral Health, The Ohio State University, 1670 Upham Drive, Suite 460G, Columbus, OH 43210 USA; 3 Department of Human Sciences, Kinesiology Program, College of Education & Human Ecology, The Ohio State University, A244 305 Annie & John Glenn Ave, Columbus, OH 43210 USA; 4 The Ohio State University, 1585 Neil Avenue, Rm. 145, Columbus, OH 43210 USA; 5 Nationwide Children’s Hospital, The Ohio State University, 700 Children’s Drive, Columbus, OH 43205 USA; 6 Department of Statistics, The Ohio State University, 404 Cockins Hall, 1958 Neil Ave, Columbus, OH 43210 USA; 7 Department of Human Sciences, Human Nutrition Program, The Ohio State University, 315 Campbell Hall, 1787 Neil Avenue, Columbus, OH 43210 USA; 8 Department of Human Sciences, Human Nutrition Program, The Ohio State University, 319 Campbell Hall, 1787 Neil Avenue, Columbus, OH 43210 USA; 9 Department of Human Sciences, Human Nutrition Program, The Ohio State University, 262B Campbell Hall, 1787 Neil Avenue, Columbus, OH 43210 USA; 10 Department of Human Sciences, Human Nutrition Program, The Ohio State University, 313 Campbell Hall, 1787 Neil Avenue, Columbus, OH 43210 USA

**Keywords:** Caregivers, Childhood obesity, Text messaging, Social media, Privacy, Summer

## Abstract

**Background:**

The most efficacious childhood obesity prevention interventions have involved caregivers directly or indirectly. Due to the high reliance on technology, research examining technological intervention approaches is warranted, particularly during the summer when parents may be more difficult to engage and the risk for excess weight gain among children is high.

**Methods:**

The feasibility and acceptability of a multi-component childhood obesity prevention intervention incorporating a caregiver component utilizing technology-based approaches—texting and social media—was explored. This was an internal pilot of the Camp Nutrition Education Recreation and Fitness (NERF) study, a group RCT for school-age children coupled to the USDA Summer Food Service Program. Feasibility and acceptability of the technology caregiver engagement component were assessed via process outcomes (participation rates) and in-depth interviews.

**Results:**

Participants (*n* = 37) were 91.9% female, 91.8% Black, 58.7% low-income, and 75.0% overweight/obese. Participation rates in texting and social media were 62.2% and < 3%, respectively. Themes emerged from the in-depth interviews were texting provides connection; desire more involvement with program; fear social media privacy intrusion.

**Conclusions:**

Results will be used to inform changes to technology-based caregiver engagement strategies to be tested in future interventions.

**Trial registration:**

Clinical Trials, NCT02908230/09-19-2016. Registered 20 September 2016

## Background

The persisting childhood obesity public health epidemic [1] demonstrates the need for expanded research focused on identification of effective obesity prevention strategies. Caregivers have a major influence on children’s health behaviors and weight status [2] through key personal affective factors (e.g., self-efficacy) and dietary and physical activity behaviors. Not surprising, the most efficacious childhood obesity prevention interventions have involved caregivers directly or indirectly [3, 4]. Past efforts utilizing traditional caregiver engagement approaches, such as in-person [5] and print media [6], have produced positive findings. However, due in part to the high reliance on technology (e.g., texting, social media), research examining these non-traditional technological approaches is warranted. Importantly, the potential for such technology approaches to serve as theoretically sound behavior change intervention strategies, either singularly or as part of a multi-component approach, for self-monitoring with immediate feedback, as well as an opportunity for support, behavioral nudging, and positive reinforcement [7], has rapidly emerged. Several literature reviews have been published highlighting the potential benefits of the use of such technologies in the specific area of childhood obesity prevention research [8–12]. However, the research is limited and none have explored the use of such caregiver targeted technologies as components of childhood obesity prevention intervention studies during the specific timeframe of summer when school is out of session and caregivers may be even more difficult to engage and child health is at a relative high risk compared to the academic months. The primary objective of this internal pilot study was to examine the feasibility and acceptability of technology-based caregiver engagement strategies (texting, social media) through assessment of process outcomes and feedback from caregiver participants.

## Methods

### Study design

This was an internal pilot of the 2015 Camp Nutrition Education Recreation and Fitness (NERF) study, an 8-week pre-test, post-test group RCT to prevent unintended, unhealthy weight gain during the summer months in underserved school-aged children [13]. Briefly, Camp NERF was a multi-component nutrition, physical activity, and mental health education intervention coupled to the US Department of Agriculture (USDA) Summer Food Service Program (SFSP), specifically open sites located at public elementary schools. Twelve eligible sites were identified and randomized (site level) to 1 of 3 programming groups: (1) Enhanced Care (nutrition, physical activity [PA], mental health, peer mentors, and caregiver engagement); (2) Standard Care (nutrition, PA); or (3) Active Control (non-nutrition, non-PA, non-mental health 4H [Head, Heart, Hands, and Health]).

Caregivers enrolled in the Enhanced Care arm of the main RCT (*n* = 37) were invited to participate in the current pilot via the baseline caregiver assessment form which was administered as part of the main RCT [13]. Technology-based approaches—texting and social media—for engaging caregivers were selected based on their appropriateness for the target population [14] and included a texting program that utilized a mass messaging platform (TextIt) [15] and social media. They were piloted during weeks 4, 5, and 6 of the 8-week intervention.

**Figure 1:**
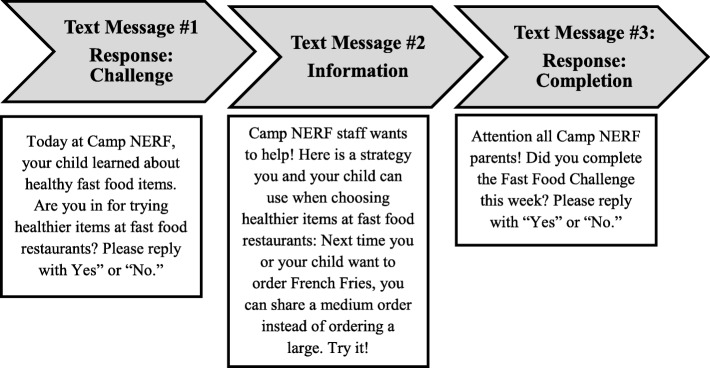
**Fig. 1** Enhanced Care Text Messaging Pilot Weekly Schematic

Text message frequency, type, and content were chosen in reference to other similar research [16, 17] and with input from local stakeholders (i.e., leaders from local government offices and non-profit organizations) and key informants (i.e., site staff) doing active work in the area of summer nutrition. Caregivers were provided the option to receive three text messages per week over the course of the study. The texts were (1) response messages (two types—challenge or completion), probing participants to reply to the text; and (2) information messages, providing nutrition or physical activity information or strategies. The first weekly message was a challenge response message, which (1) introduced the weekly nutrition or PA topic presented to their child during programming and (2) encouraged completion of a specific family nutrition goal to be attained by the end of the week. The second weekly message was an information message, which consisted of either educational information related to the topic of the week or a strategy to assist the caregivers in reaching the weekly goal. The final weekly message was a completion response message, which inquired about completion or achievement of the goal-setting challenge proposed at the start of the week. Figure 1 provides a schematic of the response (challenge or completion) and information text messages for 1-week period. Table 1 provides an overview of all text messages sent during the 3-week pilot.

**Table 1:** Enhanced Care text messaging matrix

Week	Theme	Type of message^a^	Text message
1	Fast food	Response	Today at Camp NERF, your child learned about healthy fast food items. Are you in for trying healthier items at fast food restaurants? Please reply with “Yes” or “No”.
1	Fast food	Information	Camp NERF staff wants to help! Here is a strategy you and your child can use when choosing healthier items at fast food restaurants: Next time you or your child want to order french fries, you guys can share a medium order instead of ordering a large. Try it!
1	Fast food	Response	Attention all Camp NERF parents! Did you complete the Fast Food Challenge this week? Please reply with “Yes” or “No”.
2	Screen time	Response	Happy Tuesday Camp NERF parents! Yesterday at camp, your child learned about “screen-time” and its consequences to our health. Screen-time is the total amount of time someone spends in front of any kind of screen, whether that be a TV, computer, cell-phone, or other handheld device. Doctors recommend that children should have no more than 2 hours of screen-time each day. This week, we challenge you to limit your child to 2 hours or less of screen-time. Are you in? Please reply with “Yes” or “No”.
2	Screen time	Information	Camp NERF parents, did you know that too much screen-time is also related to overweight and obesity? Here is a reason why: spending too much time sitting in front of a TV or computer screen means that we are not spending enough time being physically active, preventing our body from burning enough calories to balance the calories we eat. I hope this will encourage you to help limit your child’s screen-time this week! Do not forget to add us on Facebook: just type Camp NERF in the search bar and click “join group”!
2	Screen time	Response	Good evening Camp NERF parents! Did you complete the Screen-Time challenge this week? Please reply with “Yes” or “No”.
3	Breakfast	Response	Week 6 of Camp NERF has begun and it’s all about BREAKFAST – the importance of eating breakfast every day, and the healthy food options for a perfect kick-start to our day! So far, we have learned to watch out for cereals with extra sugar and to add fresh or frozen fruits to whole-grain cereals to give our bodies a boost of energy. This week, we challenge you to simply add some fruit to you and your child’s breakfast! Whether you give them a fresh banana, cut up strawberries to add to their cereal or oatmeal, or make a breakfast smoothie with 100% fruit juice, we encourage you to BOOST their BREAKFAST! Are you in? Please reply with “Yes” or “No”.
3	Breakfast	Information	Happy Friday Camp NERF parents! Along with learning about breakfast this week, we also learned about calcium-rich foods that are good for building STRONG bones. Did you know that many calcium-rich foods are also perfect breakfast options? - like low-fat plain milk, low-fat yogurt, and low-fat cheese! However, we can also get calcium by eating other foods, such as spinach, almonds, eggs, oatmeal, broccoli, and sunflower seeds to name a few. Whether you get your calcium from dairy or from the other items I have listed, it is still important to get calcium every day for best bone and teeth health. Good luck with this week’s challenge!
3	Breakfast	Response	Good evening Camp NERF parents! Just checking in to see how this week’s BOOST your BREAKFAST Challenge went! Did you complete the challenge? Please reply with “Yes” or “No”.

^a^Response: Text message that requires a response from the participants; Information: Text message that provides information to the participants with no option for response

Facebook and Instagram were selected as the social media platforms based on the Pew Research Foundation data which indicate that these are the most utilized outlets among low- to moderately low-income US adults [16, 18]. The nutrition topic for the week was presented on each platform and explored in greater depth by providing links to simple food recipes, news items, and recent educational articles related to the weekly topic. In addition, images or videos were added for caregivers to view and interact with counselors and other caregivers. Tables 2 and 3 provide matrices of the Facebook and Instagram posts during the 3-week pilot.

**Table 2:** Enhanced Care Facebook matrix

Week	Theme	Post description
1	Welcome	“Camp NERF - Nutrition, Education, Fitness, and Recreation - is a fun program for children grades K-5 to attend during the summer months to learn the basics of nutrition and mental health education, as well as to remain physically active while school is out! This page is for you, as parents and caregivers, to see what kinds of lessons and activities your child is participating in throughout the summer. We hope this page will encourage you to interact with your child to facilitate their learning and make this summer FUN! We are excited to meet you and to work with your child this summer!”
1	Healthy fast food choices	“Week 4 of Camp NERF is in action! This week, we learn about how to make healthy choices when eating at fast food restaurants. Although convenient, fast food is typically high in sodium, solid fats, and sugars while also lacking the essential nutrients our bodies need to function properly and remain healthy. Follow this link to read more! They even provide some tips for you and your family to consider! Check it out!”
1	Camp image highlights	Eight images of children playing with Camp NERF Counselors, including camp special guest Mr. COSI
2	Screen time	“This week at camp, we’re learning about “screen-time” and the consequences it can have on our overall health. “Screen-time” includes any amount of time spent in front of a TV, a computer, a cell-phone, or any other handheld device. Want to read more? Follow this link to read about how screen-time can affect your child’s health, and even their behavior!”
2	Healthy fast food choices	Video of children performing a rap about fast food. “We have some stars! Here are a few of our campers from [School Name] performing the “Fast Food Rap” for their peers and counsellors.”
2	Taste test wednesdays	Two images of children enjoying Taste Test Wednesdays. “Our campers love Taste Test Wednesdays! So far, we’ve tasted fruit smoothies, infused water, fruit popsicles, and yogurt parfaits. THIS WEEK we tasted roasted red pepper hummus with carrots and cucumbers! For those who don’t know, hummus is a Middle Eastern dish made from a mixture of mashed chickpeas, tahini, oil, lemon juice, and garlic. It can be used as a vegetable dip or a sandwich spread. Here are some of our campers from [School Name], tasting it and LOVING it!”
3	Healthy breakfast	“This week at Camp NERF, it’s all about BREAKFAST! Many people talk about how breakfast is the most important meal of the day, but do we know WHY? Read this article from John Hopkins School of Public Health to learn exactly why breakfast is the most important meal of the day. They even give you some tips on preparing a healthy breakfast!”
3	Visitors	Images of children interacting with adult sponsors of USDA Summer Feeding Program/Children’s Hunger Alliance/Camp NERF. “We had some visitors at Broadleigh this week! Some of our programming sponsors came to see what Camp NERF is all about!”
3	Healthy breakfast	Image of children learning how much sugar is in cereal. The grades K-2 group at [School Name] learning how much sugar is in ONE SERVING of sugary cereals, like Cocoa Puffs, Cinnamon Toast Crunch, or Frosted Flakes!

**Table 3:** Enhanced Care Instagram matrix

Week	Theme	Media description	Media caption
1	Welcome	Image of blacktop chalk writing with “Camp NERF [School Name]” and children playing in the background	Camp has officially begun! Ready to get our nutrition on #campNERF #summer2015 #nutritionrox
1	Physical activity	Video of Camp NERF counselor dancing with campers	Camp NERF [School Name] campers dancing with counselor Nikita #fun#friends #food
1	Healthy food	Image of Camp NERF camper smiling and enjoying carrots and cucumbers along with red pepper hummus as a part of Taste Test Wednesday	We love Taste Test Wednesday’s! Yesterday we tasted Roasted Red Pepper hummus with carrots and cucumbers. Participant from [School Name] loved it! #campNERF #fun #friends #food
2	Physical activity	Image of Camp NERF counselor playing basketball and shooting over the head of a jumping camper	It’s 9 am on Monday morning and our [School Name] campers are already moving! Camp NERF counselor playing basketball with camper Jordan. #summer #rocks#food #fun #friends
3	Physical activity	Video of Camp NERF counselor hula hooping with campers	Camp NERF counselor showing camper how he can HULA at [School Name] today! #GOplay #summer #rocks
3	Education/learning	Image of 2 Camp NERF counselor reading with a camper	Camp NERF counselors reading a book to camper Emanuel during free-time at [School Name] #fun #friends #food

### Participants

The target population was underserved minority children entering kindergarten through fifth grade and their adult caregiver from underserved neighborhoods in Columbus, Ohio [13].

### Data collection

Data regarding participation in the text messaging and social media platforms were retrieved from respective platform websites. Participation rates were defined as enrolling in the texting program and engaging in social media at least one time.

All caregivers at the Enhanced Care sites who agreed to participate in the pilot were invited to complete in-depth interviews at post-intervention to elicit feedback on the technology engagement strategies. Interviewers were audio-recorded, transcribed verbatim, and checked for accuracy.

Caregivers completed a demographics questionnaire and provided self-reported height and weight to the nearest inch and pound. BMI was calculated in kilogram per meter [2].

### Data analysis

For race/ethnicity, participants were classified as either Black or non-Black. Participants were classified as Black if they reported being African or African American or both African and African American and another race/ethnicity. All others were classified as non-Black. For household income, a binomial variable (low-income = 0; non-low-income = 1) was created. Annual household income data were collected categorically: (a) < $10,000; (b) $10,001–20,000; (c) $20,001–30,000; (d) $30,001–40,000; (e) $40,001–50,000; (f) $50,001–60,000; (g) $60,001–80,000; and (h) > $80,000. Based on responses to the categorical annual household income question, participants were assigned an income level based on the mid-point between the income ranges. This annual household income level was compared to the national poverty guidelines [19], and based on the number of individuals living in the household, participants were classified as low income or non-low income.

Exploratory data analyses were conducted to determine if there were any demographic differences among caregivers who engaged in the internal pilot study versus those who did not. Participation in the social media platforms was virtually non-existent, so only texting was explored. A texting engagement variable was created, and participants were assigned a value of 0 (no), 1 (low), or 2 (high). A value of 0 indicated that caregivers did not participate in text messaging; meaning, they opted out of text messaging at baseline. A value of 1 indicated that caregivers received all of the text messages but did not interact or respond to any of the response-type (challenge or completion) text messages. A value of 2 indicated that caregivers received all of the text messages and interacted or responded to at least 1 of the response-type text messages. ANOVA and chi-square tests were conducted to determine if there were any demographic differences among no-, low-, and high-texting users.

For the in-depth interviews, data analysis was guided by Grounded Theory and Interpretive Phenomonology [20, 21]. Line-by-line open coding was conducted by researchers to determine emerging themes and constant comparative analysis was employed to develop a codebook to derive focused codes from all interviews [20, 21].

The study was approved by The Ohio State University Social and Behavioral Institutional Review Board (2014B0197).

## Results

**Figure 2:**
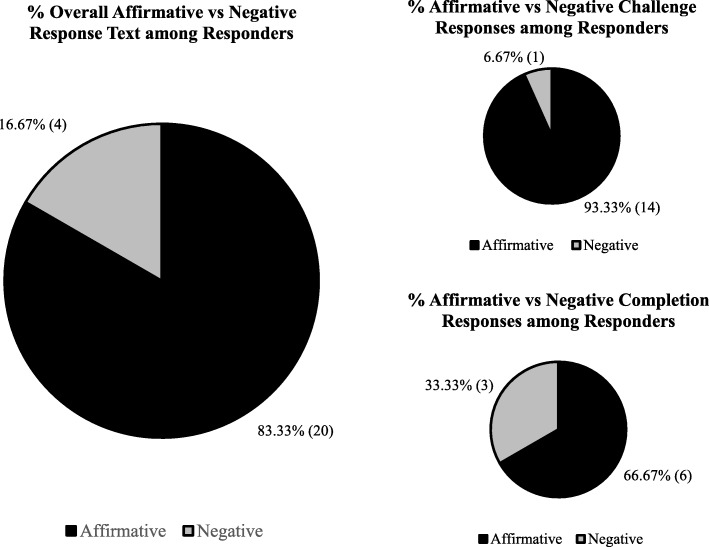
**Fig. 2** Overview of Affirmative vs. Negative Response Texts among Enhanced Care Texting Responders

Thirty-seven caregivers enrolled in the Enhanced Care arm of the main study. Descriptive summaries of participant baseline measures are presented in Table 4. Participation rates in the internal pilot study were texting—62.2% (*n* = 23); and social media—Facebook, 2.7% (*n* = 1) and Instagram, 0.0% (*n* = 0). Texting participation was further explored. One individual dropped out of the texting program after week 1. Participant characteristics did not vary by level of texting engagement (Table 4). The mean number of response texts (challenge and completion combined) received per week was 8 out of 46 sent to the 24 participants (17.4% response rate), with a higher mean challenge text response (*n* = 5 out of 23 or 21.7%) compared to the mean completion text response (*n* = 3 out of 23 or 13.0%). Of the 138 response texts sent over the course of the 3-week pilot test, the total affirmative (“yes”) responses to accept or complete the challenge was *n* = 20 out of 24 received (83.3%) and higher for the challenge texts (*n* = 14 out of 15 or 93.3%) compared to the completion texts (*n* = 6 out of 9 or 66.7%) (Fig. 2).

**Table 4:** Enhanced Care caregiver demographics by participation in the texting program

	Total sample (*n* = 37)	None (*n* = 14)	Low (*n* = 13)	High (*n* = 10)	*P* value
Age, mean ± SE	37.11 ± 1.82	39.9 ± 3.5	37.6 ± 3.1	32.9 ± 2.1	0.31^a^
Gender, % (*n*)					
Male	8.11% (3)	14.3% (2)	7.7% (1)	0.0% (0)	0.47^b^
Female	91.89% (34)	85.7% (12)	92.3% (12)	100.0% (10)	
Race, % (*n*)					
Black	91.76% (31)	83.3% (10)	100.0% (12)	90.0% (9)	0.35^b^
Non-Black	8.82% (3)	16.7% (2)	0.0% (0)	10.0% (1)	
Income, % (*n*)					
Low-income	77.42% (24)	90.0% (9)	58.3% (7)	88.9% (8)	0.31^b^
Non-low-income	22.58% (7)	10.0% (1)	41.7 (5)	11.1% (1)	
BMI, mean ± SE	29.91 ± 1.25	27.7 ± 1.6	32.4 ± 2.5	28.9 ± 1.7	0.27^a^
BMI Classification, % (*n*)					
Underweight	3.57% (1)	11.1% (1)	0.0% (0)	0.0% (0)	0.33^b^
Normal	2.14% (6)	22.2% (2)	9.1% (1)	37.5% (3)	
Overweight	32.14% (9)	33.3% (3)	36.4% (4)	25.0% (2)	
Obese	42.86% (12)	33.3% (3)	54.5% (6)	37.5% (3)	

^a^ANOVA

^b^Chi-square

Three main themes emerged through the in-depth interview data analysis. Caregiver feedback (*n* = 3) revealed a perceived connection to camp programing through text messaging, a desire for increased caregiver interaction with daily camp activities, and a perceived potential intrusion of privacy via social media use.

### Perceived connection to program via texting

Participants conveyed that texting provided them with insight and connection to the camp activities and lessons. Caregivers were able to reinforce camp lessons through healthy behavior modifications at home prompted by the weekly challenge and informational messages. Participants were also able to engage other family members not enrolled in the camp and encourage them to participate in the positive behavior changes.

Yeah like I said I think it was good cause then like it just makes you kinda consciously aware of you know trying to reinforce- especially over the summer time- and implement some things that you may do throughout the year but in the summer it’s kinda like free for all, eat cereal and grab a sandwich. [Female; age, 39]

Yeah it helped me with the other kids too that wasn’t in the program… So we got to do more activities at home than doing unhealthy. [Female; age, 28]

Participants expressed that the text messages provided caregivers with an undemanding, virtual connection to the camp activities. Receiving and replying to text messages was less involved than requiring caregivers to physically come to the camp setting. Additionally, the text messaging platform allowed participants to respond on their own time as their schedule permitted without demanding an immediate response like a phone call requires.

Text messaging and Facebook are good ways to get people involved, if they don’t wanna be here in person. [Female; age, 28]

Sometimes I do not feel well and I do not answer my phone so that was, that was okay to me. That was perfectly fine. [Female; age, 38]

Participants were asked to provide details regarding what they enjoyed about the text messaging platform. All participants specifically referenced the weekly challenge messages prompting caregivers to implement a healthy suggestion as a positive component of the intervention. The challenge messages motivated and encouraged participants to try and apply new habits and activities in their home environment.

Yeah, that was a challenge. Something like we had never done so it was nice to do something new… I was like “oh alright let’s try this this week”. [Female; age, 28]

And so- and then it made me think about some stuff- it was like “do I really- could I really control like family dinner time and TV time and all that? [Female; age, 39]

I think it was one week that was a lesson uhm swap fast food for uhm, for uh, healthier meals, yeah, so I did that. [Female; age, 38]

All participants agreed that three text message matrices per week were an acceptable and sufficient amount of weekly contacts. The spacing between messages provided adequate time to consciously think about and implement healthy suggestions without burdening participants with overly frequent contacts.

Yeah it wasn’t too much, it wasnt too little. And then was just like- ah you kinda look forward to it because it’s kinda like- it makes you think about some stuff like okay let me make sure that we are having dinner, you know we are discussing like what we are doing. I mean, we do that anyway but you know it kinda puts in your su- in your conscious mind… I woulda disliked it if it was too many. But it wasn’t so it was good. [Female; age, 39]

A few participants commented on the simplistic and unintrusive nature of the text messaging intervention. One participant noted that the messages provided quick and easy access to healthy habit suggestions without delivering an overwhelming amount of content. Participants also proposed that text messages were less invasive than other forms of contact such as weekly phone calls or daily emails from camp personnel.

...I liked the text messages because it’s easy. And sometime I would not see it [sic] and I was like oh! I was like- and then there were yes, no questions like to where it wasn’t ya know all along and drawn out. [Female; age, 39]

No I believe they were fine. Helpful- you know not like overly invasive or somethin’ where someone would call you and be like okay this is Camp NERF this week. [Female; age, 38]

And be like you know you subscribe to some email and it’s like every day are you kidding me? [Female; Age. 39]

Participants were asked to reveal what they disliked about the text messaging intervention and provide suggestions for improvement. Multiple participants expressed a dislike of not being prompted to respond to all three weekly message matrices. Participants recommended utilizing a more interactive messaging platform with more opportunities to reply and participate in challenges.

That you could not reply back. [Female; age, 28]

...more responses. I mean cause some of the questions were yes, no, but then it was like I do not know I thought it was maybe like a question in like another part that I couldn’t respond to… I would’ve liked to have responded... [Female; age, 38]

### Desire for increased caregiver interaction with daily camp activities

In addition to text messaging, participants also approved of a social media presence. Multiple participants perceived the Camp NERF social media platforms as informative means of learning what the children are doing during the day at camp.

I just like that y’all had a website. And it’s like some other programs does not [sic] have anything so you do not get to know really more about it. So I was happy that y’all had like a instagram [sic] and a Facebook. But I did not dislike nothin’ [sic]. [Female; age, 28]

A participant expressed a desire for copies of camp itineraries, including breakfast and lunch menus. Increased caregiver knowledge regarding the daily camp menus may offer ideas for healthy family meals at home and reinforce lessons taught at camp.

I do not think that would deter the kids, if anything I believe it should like you know have the parents like oh yeah lets [sic] go get you a salad or whatever like some healthy foods and yer’ it’s better than runnin’ in the house, runnin’ to the cabinet and grabbin’ a-a- a little Debbie cake or you know somethin’ like that… I would- I would- definitely seein’ a menu or somethin’. [Female; age, 38]

Another participant suggested offering more activities to involve caregivers and other family members not enrolled in the camp. Family participation may improve self-efficacy and social support for making healthy decisions outside of the camp setting.

...maybe like once a week have like a little family gathering like when y’all do the meals and stuff… and then that could be another challenge at home that they could try to do too. With the text message they sending out [sic]. [Female; age, 28]

### Perceived intrusion of privacy via social media and barriers to social media use

While caregivers supported using social media for viewing information about camp lessons and activities, participants expressed concern about a potential invasion of privacy. Caregivers may fear judgment about their child-rearing style based on their personal social media account posts, or they may feel unsafe sharing information about their family with other participants and camp personnel.

Yeah, yeah. Yeah that’s why I gave the other account cause I am like they are gonna see some stuff- like no! [laughs] [Female; age, 39]

Another participant noted that their personal social media account does not use their actual name. Participants using pseudonym accounts may be difficult to reach and invite to join camp social media websites. Additionally, camp personnel may not approve of pseudonym accounts accessing the camp websites in order to protect the confidentiality of camp participants.

Probably not because my account does not have my name, [Female; age, 38]

## Discussion

The main objective of the current study was to assess the feasibility and acceptability of the technology caregiver engagement strategies through assessment of key process outcomes (rates of participation) and by eliciting feedback from intervention caregiver participants via in-depth interviews. This was an internal pilot study that occurred as part of a larger RCT (Camp NERF 2015), in which child weight status (i.e., BMI *z*-score) was the primary outcome of interest [13]. Due to the multi-level nature of Camp NERF intervention, the objectives of main RCT extended beyond the child to include assessment of caregivers (i.e., self-efficacy, physical activity, and BMI), though are not presented here given the nature of the publication (i.e., pilot and feasibility studies) [13].

Results demonstrated mixed findings in that participation in the social media component was low (< 3% participation rate, low feasibility; comparatively low to similar research [22]) compared to the texting program component (62% participation rate, high feasibility; comparatively high compared to similar work [23]). The relative low participation rates for Facebook and Instagram could be due to the issue of participants not having accounts with the two platforms; however, it is not possible to confirm this with the data collected. It may also be attributed to differences in recruitment efforts and inherent differences between the technologies (social media, texting). For the texting program, participants who indicated during the baseline form that they wanted to participate were automatically enrolled in the program through the TextIt [15] platform. For the Facebook and Instagram programs, participants were invited to the Camp NERF pages through the social media platforms, but would have to log-on, accept the invitations, and actively engage. Data from the in-depth interviews revealed that there was difficulty in locating and inviting caregivers to participate in the Camp NERF pages due to participants using pseudonym accounts. To uphold participant confidentially, future program social media pages may not allow participants with pseudonym accounts to become members of the page thus limiting caregiver engagement. Additionally, themes derived from the in-depth interviews indicated a privacy concern among caregivers. Caregivers were apprehensive about the visibility of their social media profile information and personal posts. This concern may be explained by caregivers feeling unsafe sharing information about their families and children with other members of the camp page that they may not know or trust. Additionally, caregivers may fear judgment and evaluation of their lifestyle or child-rearing methods from other caregivers or camp personnel. Both a fear of sharing personal information and receiving judgment from others may have contributed to the lack of participation in Enhanced Care social media platforms. These results point to the critical need for caregiver engagement technology components in future iterations of Camp NERF and other similar childhood obesity prevention interventions that include feedback from the target-audience (e.g., identification of strategies to overcome the fear of privacy breaches on social media to improve caregiver engagement) for optimal intervention tailoring and effectiveness. Adapting “lessons learned” from other similar research will also be important (e.g., making the social media website private, including a video-based curriculum) [22].

Due to the higher participation in the texting program component, a higher volume of process data was collected relative to the social media component. Analysis of texting program process data revealed that those who responded to a text, were more likely to respond to the “Accept the challenge” vs “Complete the challenge” text, and more likely to provide a “Yes” vs “No” response to the “Accept the challenge” vs “Complete the challenge” text. Results may imply that caregivers are more willing to accept health challenges, and less willing to complete such health-oriented challenges [24]. Future research should focus on ways to increase completion of health challenges among caregivers, such as utilization of a mobile app in addition to texting [25]. Also, analyses revealed that those who responded to a text of either type text (“Accept the challenge” vs “Complete the challenge”) were more likely to give a “Yes” vs “No”, which may imply that people desire to be engaged with the intervention activities, which corroborates with the data generated from the in-depth interviews that participants enjoyed the texting piece of the intervention because it made them feel connected to the program. On the other, these data could indicate a potential social desirability bias [26]. Future research should continue to include qualitative assessments (i.e., in-depth interviews) to better understand the underlying reason(s) the increased probability of providing an affirmative text response.

Strengths of the current study include the mixed-methods approach to data collection, which provided the ability to probe deeper into the trends observed in the (quantitative) data. In addition, the collection of process outcomes data for the technology components allowed the opportunity to determine responsiveness to the techniques. The primary study limitation relates to the timing of the pilot. Relative to the main 8-week RCT, it was conducted during weeks 4 to 6. Ideally, this pilot work have occurred prior to the main RCT, however, this was not possible given the time constraints relating to funding. In addition, there were challenges in recruiting caregivers, a common issue at USDA SFSP open sites where caregivers are not required to accompany their children to the program. Regardless, this work is critical for informing changes to technology-based caregiver engagement strategies to be tested in future interventions. Another limitation is the low response rate for the in-depth interviews. It will be imperative for future efforts to increase the number of participants interviewed to confirm that the themes identified here are in fact true.

## Conclusions

Results from this study may be utilized to improve caregiver engagement strategies in the delivery of future iterations of Camp NERF and other similar health behavior interventions for underserved school-age children during the summer months, including development of tailored strategies to increase participation in the social media component (e.g., addressing issue of privacy concerns, match the ease with which participants are enrolled in social media to texting) and increase the number of caregivers who complete health challenges (e.g., addition of mobile app technology).
